# Trends and results in treatment of gastric cancer over last two decades at single East European centre: a cohort study

**DOI:** 10.1186/1471-2482-14-98

**Published:** 2014-11-26

**Authors:** Antanas Mickevicius, Povilas Ignatavicius, Rytis Markelis, Audrius Parseliunas, Dainora Butkute, Mindaugas Kiudelis, Zilvinas Endzinas, Almantas Maleckas, Zilvinas Dambrauskas

**Affiliations:** Department of Surgery, Lithuanian University of Health Sciences, Eiveniu Str. 2, Kaunas, LT-50009 Lithuania; Laboratory of Surgical Gastroenterology, Institute for Digestive System Research, Lithuanian University of Health Sciences, Eivenių str. 2, Kaunas, LT-50009 Lithuania; Department of Oncology, Lithuanian University of Health Sciences, Eivenių str. 2, Kaunas, LT-50009 Lithuania

**Keywords:** Gastric cancer, Complications, Survival, Mortality

## Abstract

**Background:**

A steady decline in gastric cancer mortality rate over the last few decades is observed in Western Europe. However it is still not clear if this trend applies to Eastern Europe where high incidence rate of gastric cancer is observed.

**Methods:**

This was a retrospective non-randomized, single center, cohort study. During the study period 557 consecutive patients diagnosed with gastric cancer in which curative operation was performed met the inclusion criteria. The study population was divided into two groups according to two equal time periods: 01-01-1994 – 31-12-2000 (Group I – 273 patients) and 01-01-2001 – 31-12-2007 (Group II – 284 patients). Primary (five-year survival rate) and secondary (postoperative complications, 30-day mortality rate and length of hospital stay) endpoints were evaluated and compared.

**Results:**

Rate of postoperative complications was similar between the groups, except for Grade III (Clavien-Dindo grading system for the classification of surgical complications) complications that were observed at significantly lower rates in Group II (26 (9.5%) vs. 11 (3.9%), p = 0.02). Length of hospital stay was significantly (p = 0.001) shorter (22.6 ± 28.9 vs. 16.2 ± 17.01 days) and 30-day mortality was significantly (p = 0.02) lower (15 (5.5%) vs. 4 (1.4%)) in Group II. Similar rates of gastric cancer related mortality were observed in both groups (92.3% vs. 90.7%). However survival analysis revealed significantly (p = 0.02) better overall 5-year survival rate in Group II (35.6%, 101 of 284) than in Group I (23.4%, 64 of 273). There was no difference in 5-year survival rate when comparing different TNM stages.

**Conclusions:**

Gastric cancer treatment results remain poor despite decreasing early postoperative mortality rates, shortening hospital stay and improved overall survival over the time. Prognosis of treatment of gastric cancer depends mainly on the stage of the disease. Absence of screening programs and lack of clinical symptoms in early stages of gastric cancer lead to circumstances when most of the patients presenting with advanced stage of the disease can expect a median survival of less than 30 months even after surgery with curative intent.

## Background

Although a steady decline in gastric cancer mortality rates over the last few decades is observed, gastric cancer still remains the fourth most common cancer and is the second leading cause of cancer death worldwide with poor survival rates [1]. While incidence rates of gastric cancer in North America, Africa, South and West Asia are declining, rates in North-East Asia, Eastern part of South America and Eastern Europe stay high [1–3]. Surgery remains the major and potentially curative treatment method for resectable gastric cancer. Considering the location and size of the tumor as well as invasion to the adjacent organs, routinely standard radical total or subtotal gastrectomy with lymphadenectomy or multiorgan resections are performed [4–6]. The overall 5-year survival rate of patients with advanced resectable gastric cancer differs between different countries and different centres, but in general it ranges from 10% to 30% [5, 7, 8]. Previous studies have shown that age, lymph node and liver metastasis, disease stage and tumour size are important predictive factors for survival in patients with resectable gastric cancer [9–11]. However it is not certainly clear if these predictive factors are the same in all regions and why incidence rates of gastric cancer are still high in the region of Eastern Europe.

The aim of this single centre study was to compare the clinical course and outcomes, such as postoperative complications, the length of hospital stay and mortality rate, over two distinctive time periods.

## Methods

This was a retrospective non-randomized, single center, cohort study. Data collection was performed at the Department of Surgery, Lithuanian University of Health Sciences using specially developed and maintained database from 01-01-1994 to 31-12-2007. During this period 708 patients underwent radical gastrectomy. Five hundred fifty seven consecutive patients were included in the study according to the following inclusion criteria: (1) histologically proven gastric adenocarcinoma; (2) diagnosis based on the UICC TNM staging classification; (3) curative D1 or D2 gastrectomy performed; (4) available complete medical record; (5) postoperative follow-up. Patients with proven distant metastatic disease and in whom only palliative surgery was performed, were excluded from the study. The study population was divided in two groups according to two equal time periods: 01-01-1994 – 31-12-2000 (Group I – 273 patients) and 01-01-2001 – 31-12-2007 (Group II – 284 patients). During the first time period patients diagnosed with gastric cancer were treated according to the guidelines of that time. Standardized protocol was introduced in the year 2001: preoperative evaluation and care (preoperative computed tomography (CT) staging, prophylactic antibiotics), surgical treatment and postoperative care (prophylaxis of thromboembolic disorders; early mobilisation; on day 2 after surgery patients were allowed to drink clear liquids; on postoperative day 3 the soft diet was allowed; drain’s placement was at the discretion of the surgeon). The Kaunas Regional Biomedical Research Ethics Committee approved the study (protocol no. BE-2-10) and allowed the use of publicly unavailable database. All patients provided written informed consent. The primary outcome was measured as the five-year survival rate. The gastric cancer- related survival, rates of postoperative complications, the length of hospital stay and 30-day mortality rate were considered as secondary outcomes. The outcomes were studied to evaluate the progress in gastric cancer treatment results over time.

### Surgical procedure

All the surgical procedures were based on the intention to cure. The extent of the surgical procedure was planned based on pre-operative and intra-operative findings, physical condition of the patient. Considering the location of the tumor, routinely standard total (adenocarcinoma involving the proximal third of the stomach) or subtotal (adenocarcinoma of the distal and middle thirds of the stomach) gastrectomy with D1 or D2 lymphadenectomy and a Roux-en-Y reconstruction was performed. Surgical procedures and the definition of lymphadenectomy referred to the Japanese Classification of Gastric Carcinoma [12]. Combined multiorgan resections were performed in cases of advanced tumors involving the pancreas, colon or spleen. Surgery was considered as a curative when there was no macroscopically residual tumor after surgery and resection margins were histologically clear (R0).

### Postoperative course

Postoperative complications were classified according Clavien-Dindo grading system for the classification of surgical complications (Grade I - V) [13].

Different mainly fluorouracil (5-FU) based adjuvant chemotherapy regiments were inconsistently used postoperatively in the period from 1994 to 2000. Whereas patients during the period from 2001 to 2007 as a standard received a combined 5-FU and leucovorin adjuvant chemotherapy or concurrent chemoradion treatment (5-FU and leucovorin with 45 Gy radiation dose) in more advanced cancer cases.

### Statistical analysis

Statistical analysis was performed using SPSS 14.0 for Windows *(SPSS Inc., Chicago, USA)*. The data are presented as mean ± Standard deviation or median and range. The cumulative survival was determined by the Kaplan-Meier method, and univariate comparisons between the groups were performed using the log-rank test. The independent prognostic factors were examined by Cox regression analysis. For comparison between groups, the Mann–Whitney test or Student’s t test were employed where appropriate. P < 0.05 was considered statistically significant.

## Results

**Figure 1:**
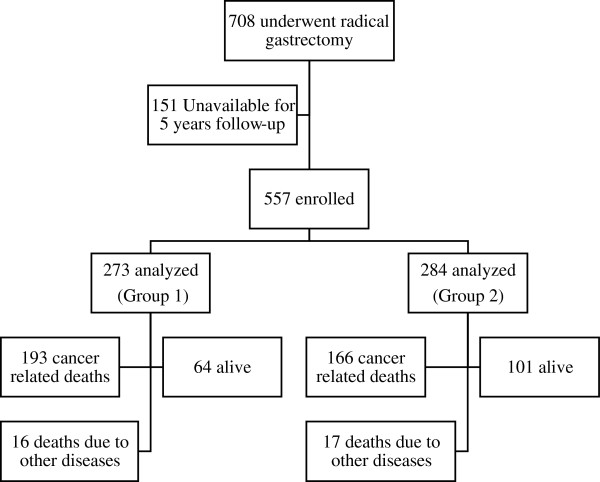
**Patient’s distribution between groups and subgroups.**

Seven hundred and eight patients with proven gastric adenocarcinoma underwent a subtotal or total gastrectomy and D1 or D2 lymphadenectomy with curative intent between 1994 and 2007. One hundred fifty one patients were unavailable for 5 years follow-up. The most frequent detected reason of the unavailability was moving abroad. Data from 557 patients which were followed-up postoperatively was analysed. The distribution of patients between groups and subgroups is shown in Figure 1. There were no significant differences between the groups in gender and age. The number of elderly (>65 years) patients was also similar (50.5% vs. 52.8%). Total gastrectomy was statistically significantly more often performed in Group I (29.3%) than in Group II (18.3%). D2 lymphadenectomy was more often performed than D1 lymphadenectomy in both groups (Table 1). Gastric cancer in early stages (IA - IIA) was more frequently diagnosed in Group II than in Group I and in late stages (IIB - IIIC) more frequently in Group I. Statistically significant difference was found only when comparing stages IIIB-IIIC. Significantly more patients were diagnosed with lower stage of the primary tumor (T stage) in Group II (13.7% vs 7.7% in T1 stage (P = 0.04) and 35.6% vs 15.4% in T2 stage (P = 0.0001)). On the contrary in Group I more patients were diagnosed in T4 stage (33.7% vs 9.9%, P = 0.0001) (Table 2).

**Table 1:** **Patients’ characteristics**

Parameter	Group I	Group II	P value
**Gender**			
Male	155 (56.8%)	164 (57.7%)	0.944
Female	118 (43.2%)	120 (42.3%)	0.938
**Age**	63.2 ± 12.7	64.3 ± 12.1	0.331
**≤** 65	135 (49.5%)	134 (47.2%)	0.767
> 65	138 (50.5%)	150 (52.8%)	0.772
**Procedure**			
Total gastrectomy	80 (29.3%)	52 (18.3%)	0.019
Subtotal gastrectomy	193 (70.7%)	232 (81.7%)	0.273
D1 lymphadenectomy	7 (2.6%)	5 (1.8%)	0.572
D2 lymphadenectomy	266 (97.4%)	279 (98.2%)	0.952
**Total**	273	284	

**Table 2:** **Staging of the disease**

Parameter	Group I	Group II	P value
**Pathological stage**			
T1	21 (7.7%)	39 (13.7%)	0.042
T2	42 (15.4%)	101 (35.6%)	0.0001
T3	118 (43.2%)	116 (40.8%)	0.775
T4	92 (33.7%)	28 (9.9%)	0.0001
**N stage**			
N0	86 (31.5%)	91 (32.0%)	0.931
N1	68 (24.9%)	87 (30.6%)	0.276
N2	92 (33.7%)	73 (25.7%)	0.133
N3	27 (9.9%)	33 (11.6%)	0.589
**TNM Stage**			
IA	18 (6.6%)	30 (10.6%)	0.135
IB	30 (11.0%)	47 (16.5%)	0.114
IIA	43 (15.8%)	61 (21.5%)	0.165
IIB	51 (18.7%)	47 (16.5%)	0.586
IIIA	58 (21.2%)	65 (22.9%)	0.765
IIIB	51 (18.7%)	24 (8.5%)	0.002
IIIC	22 (8.1%)	10 (3.5%)	0.044

When analyzing postoperative course of the disease shorter hospital stay (16.20 ± 17.01 vs. 22.61 ± 28.96 days, P = 0.001) and lower 30-day mortality rate (1.4% vs 5.5%, P = 0.0173) was identified in Group II. During postoperative period in 6 patients (2.2%) of Group I and in 7 patients (2.5%) of Group II anastomotic leakage was identified. However grade of postoperative complications was similar between both groups. Only Grade III (Complications requiring surgical, endoscopic or radiological intervention) complications were statistically significant more often identified in Group I (9.5% vs 3.9%, P = 0.017) (Table 3).

**Table 3:** **Postoperative course and outcomes**

Parameter	Group I	Group II	P value
**Hospital stay** (days)	22.61 ± 28.96	16.20 ± 17.01	0.001
Complications*	55 (20.1%)	42 (14.8%)	0.187
I	13 (4.7%)	15 (5.4%)	0.848
II	8 (2.9%)	6 (2.1%)	0.598
III	26 (9.5%)	11 (3.9%)	0.017
IV	4 (1.5%)	5 (1.7%)	1.000
V	4 (1.5%)	5 (1.7%)	1.000
**30-day mortality**	15 (5.5%)	4 (1.4%)	0.017

*- According to Clavien-Dindo grading system for the classification of surgical complications.

The survival analysis revealed higher median overall survival (months) in Group I (48.40 ± 65.966 vs. 43.78 ± 39.736). However Group I patients are observed for a longer time period and long-term survivors among them could influence this outcome. In contrary when analysing 1-year and 5-year survival rates, significantly higher survival is observed in Group II (71.5% vs. 50.2% and 35.6% vs. 23.4%). Patients with more advanced T stage and involved lymphnodes had worse 5-year survival prognosis as compared with patients with les advanced T stage and no lymphnodes involvement. However statistically significant difference was found only when analyzing lymphnodes involvement (p < 0.05).

**Figure 2:**
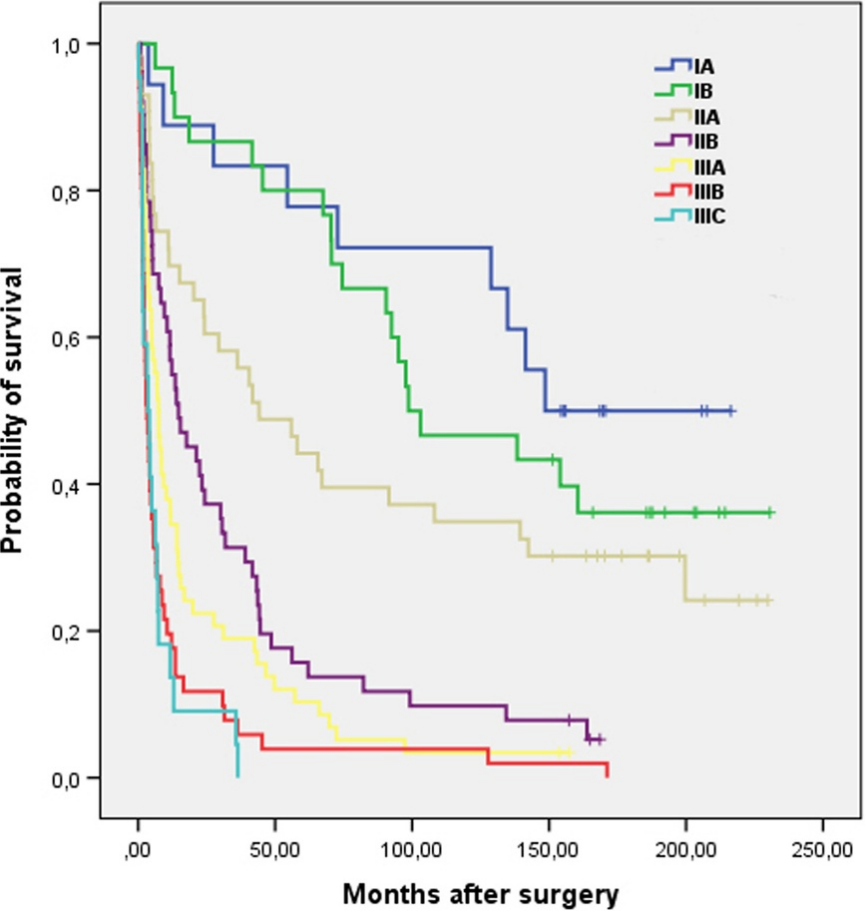
**Overall survival (in months) after surgery according to TNM stage (Group I).**

**Figure 3:**
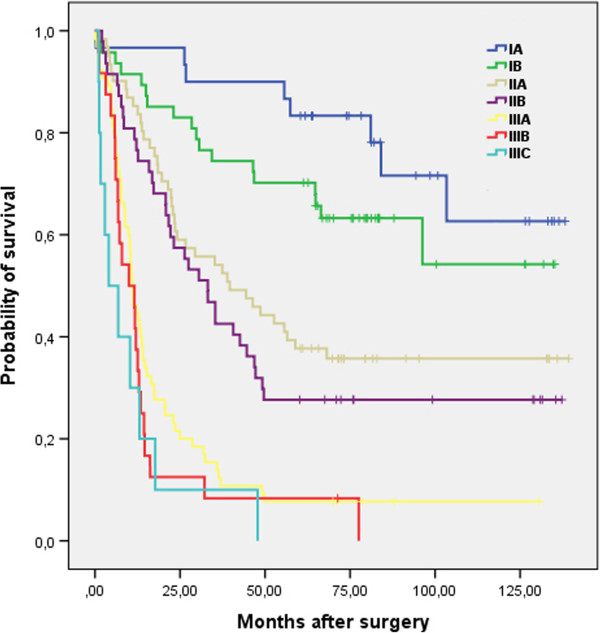
**Overall survival (in months) after surgery according to TNM stage (Group II).**

In both groups the reasons of death were similar; the majority of patients died of gastric cancer (92.3% vs. 90.7%). There was no difference in 5-year survival rate when comparing different TNM stages between both groups (Table 4). However in early stages (IA - IIA) survival rate was higher comparing with advanced stages (IIB - IIIC) (Figures 2 and 3).

**Table 4:** **Postoperative survival analysis**

Parameter	Group I	Group II	P value
**Overall survival** (months)	48.40+/-65,966	43.78+/- 39.736	0.319
**1-year survival rate**	137 (50.2%)	203 (71.5%)	0.013
**2-years survival rate**	110 (40.3%)	150 (52.8%)	0.083
**5-year survival rate**	64 (23.4%)	101 (35.6%)	0.021
IA	11 (61.1%)	25 (83.3%)	0.644
IB	22 (73.3%)	33 (70.2%)	1.000
IIA	17 (39.5%)	23 (37.7%)	1.000
IIB	7 (13.7%)	13 (27.7%)	0.221
IIIA	7 (12.1%)	5 (7.7%)	0.552
IIIB	0	2 (8.3%)	0.111
IIIC	0	0	-
**Deaths**	209 (76.6%)	183 (64.4%)	0.210
Gastric cancer related	193 (92.3%)	166 (90.7%)	0.942
Other causes	16 (7.7%)	17 (9.3%)	0.717

## Discussions

The incidence of gastric cancer in Eastern Europe remains high following Eastern Asia and South America. The highest gastric cancer incidence rate in European Union (EU) is reported in Lithuania [14]. In this single-institutional study we present case series from large university hospital in Lithuania raising a question: is there a significant progress in terms of treatment success and survival among patients diagnosed with gastric cancer and treated with curative surgery over time? To clarify changes, positive or negative tendencies the two groups on background of time of surgery were created.

Most of demographic data, clinicopathologic characteristics in our study are comparable to the groups presented from other European countries. Regrettably, gastric cancer remains often diagnosed in advanced stages in Lithuania, leading to poor prognosis. Late diagnosis of gastric cancer is a well-known problem among patients from Western countries. Hundahl et al. [7] from United States (US) report 65% of gastric cancers presenting at an advanced stage (T3-T4) with a nearly 85% of tumours accompanied with lymph node affection at the time of surgery. The data are very close to ours (T3-T4 - 63.6%; N+ 68.2%). However higher gastric cancer incidence rate in Lithuania leads to even more actual problem.

The interesting difference identified between our data and studies done in Western Europe - a lack of growing incidence of upper-third gastric cancer. In contrary, we even had more distal and middle third tumours and higher proportion of patients underwent subtotal gastrectomy in Group II. Although we have not analysed this factor in our study, high prevalence of Helicobacter pylori infection in Lithuania (78.5% in the year 1999 and 69.7% in the year 2005) could be related to the high incidence of gastric cancer [15].

The overall incidence of directly surgery-related postoperative complications in our study (anastomosis or duodenal stump leakage) <3% is comparable to majority of published data [16–18]. The rate and grade of postoperative complications (except Grade III complications) in our study was similar in both groups; however 30-day mortality rate and in-hospital stay decreased significantly in Group II. These results should be considered as a consequence of more detailed preoperative patients’ selection, standardised surgery technique, and improved perioperative care over time.

The overall 5-year survival rate was slightly higher in Group II, however remaining below 40% in entire cohort. The higher survival rate possibly caused by a higher rate of early detection (standardized protocol of diagnosis) of gastric cancer (more T1, T2 tumours, less IIIB, IIIC stages in Group II), perioperative care improvement over time and possibilities of palliation procedures in cases of recurrent disease. The most common cause of treatment failure in our study was peritoneal recurrence and spread of the disease. Similar data are presented by other authors [19, 20]. Observed 5-year survival rate in early stages (IA 61.1% vs. 83.3% and IB 73.3% vs. 70.2%) of gastric cancer is lower than in Eastern countries, but is similar to the data presented by the Western European countries from the similar time period [21, 22].

Regarding the surgical technique and extent of lymph node dissection, it became highly standardised over last 15 years. The patients in our study mainly underwent gastrectomy with a Roux-en-Y reconstruction and D2 lymphadenectomy (97.4% vs. 98.2%). There has been controversy regarding the extent of lymph node dissection around the European centres in last decade, pointing on higher postoperative morbidity after D2 dissection, however most experts suggest that extended lymphadenectomy could be performed with acceptable morbidity and mortality rate by specialized surgeons in large-volume centres [23–25].

## Conclusions

Despite some positive changes in early postoperative mortality rate, hospital stay and overall survival over the time, gastric cancer treatment results remain poor. Prognosis of treatment of gastric cancer depends mainly on the stage of the disease. Absence of screening programs and lack of clinical symptoms in early stages of gastric cancer lead to circumstances when most of the patients presenting with advanced stage of the disease can expect a median survival of less than 30 months even after curative intent surgery. The most efficient way to reach more significant progress in gastric cancer treatment should concentrate mostly on earlier diagnosis, when survival results after radical surgery are far more promising.
